# Treatment of Recurrent Focal Segmental Glomerulosclerosis in Pediatric Kidney Transplant Recipients: Effect of Rituximab

**DOI:** 10.1155/2011/389542

**Published:** 2011-04-26

**Authors:** Christine Sethna, Corinne Benchimol, Hilary Hotchkiss, Rachel Frank, Lulette Infante, Suzanne Vento, Howard Trachtman

**Affiliations:** ^1^Division of Nephrology, Department of Pediatrics, Cohen Children's Medical Center of New York, 269-01 76th Avenue, New Hyde Park, NY 11040, USA; ^2^Division of Pediatric Nephrology, Department of Pediatrics, Mount Sinai Medical Center, New York, NY 10029, USA

## Abstract

Recurrence of focal segmental glomerulosclerosis (FSGS) after renal transplantation is a complication that often leads to graft loss. There is no consensus on the optimal treatment of recurrent FSGS. Rituximab, a monoclonal antibody to CD20, may be a useful treatment of this complication. *Methods*. We report four pediatric cases of recurrent FSGS treated with rituximab and plasmapheresis. *Results*. Four children (2M/2F), age 15.3 ± 2.6, with recurrent FSGS posttransplant were identified. Four doses of rituximab were administered 171 ± 180 days posttransplant and 114 ± 169 days after the start of plasmapheresis. Three children responded with complete remission, one of whom relapsed after four months. One child had a partial response with a decrease in proteinuria that was not sustained. No adverse side effects were reported during treatment or followup (mean 22.5 months). *Conclusions*. Rituximab is a safe and well-tolerated ancillary treatment for recurrent FSGS in pediatric patients in conjunction with plasmapheresis.

## 1. Introduction

Recurrence of focal segmental glomerulosclerosis (FSGS) occurs in 25–53% of patients with this glomerulopathy who receive a kidney transplant and in over 80% of patients receiving subsequent transplants after previous recurrence [1–6]. Recurrent disease often leads to graft loss [7]. The risk of recurrence appears to be higher in pediatric kidney transplant recipients with FSGS compared to adult patients [4, 8]. 

FSGS can recur as early as a few hours after transplant and as late as two years post-transplant [7]. The pathogenesis of this entity is not completely understood; however, glomerular injury is thought to be mediated by a low-molecular weight circulating permeability factor that affects podocyte function or by loss of an inhibitor of this factor. Plasmapheresis (PP) has been shown to decrease activity of the permeability factor in the circulation and to induce remission of recurrent FSGS, thus supporting a key role of the permeability factor in the pathogenesis of this disease [2]. 

There is no consensus on the optimal treatment of recurrent FSGS due to the lack of controlled studies. Several case series report complete remission rates of 50–67% in pediatric transplant recipients treated with PP [9–11]. Other therapies including administration of high doses of calcineurin inhibitors, cyclophosphamide and angiotensin converting enzyme inhibitors have been tried with variable results. There are scattered reports that rituximab, a monoclonal antibody to CD20, may be useful for the treatment of this complication. 

We report our experience of treatment with rituximab and PP in an unselected group of pediatric renal transplant recipients with recurrent FSGS at a single center.

## 2. Materials and Methods

Medical records were retrospectively reviewed to identify children who received a kidney transplant at the Mount Sinai Medical Center and who developed recurrence of FSGS that was treated with rituximab during the past 2 years. Recurrence of FSGS was diagnosed based on the presence of nephrotic-range proteinuria in the absence of another cause and a decline in the serum albumin concentration. Proteinuria was measured by the protein (mg/dL) to creatinine (mg/dL) ratio (U_P/C_) in a first morning urine sample with nephrotic-range proteinuria defined as >2.0. 

Once recurrence of FSGS was documented, PP was prescribed as clinically indicated. Rituximab was administered intravenously at a dose of 375 mg/m^2^ once a week for four weeks. The initial dose of rituximab was administered in the inpatient setting. The infusion was started at a rate of 50 mg/hour and was increased by 50 mg/hour increments as tolerated every 30 minutes, to a maximum rate of 400 mg/hour. Subsequent infusions were started at 100 mg/hour and increased by 100 mg/hour increments as tolerated every 30 minutes, to a maximum rate of 400 mg/hour. All patients were premedicated with acetaminophen and diphenhydramine prior to each dose of rituximab. The response to therapy was measured by serial U_P/C_ ratios in the first morning urine sample.

## 3. Results

A total of four children (two males and two females) age 15.3 ± 2.6 (range 13–18), were identified with recurrent FSGS and who were treated with rituximab. All children had intact native kidneys. They all received deceased donor allografts and an immunosuppressive regimen that consisted of thymoglobulin induction, prednisone, tacrolimus, and mycophenolate mofetil. Renal biopsy confirming FSGS recurrence was performed in three children (all but Case 2). PP was initiated 58 ± 106 days post-transplant (range 2–217 days). Rituximab was administered 171 ± 180 days (range 10–395 days) post-transplant and 114 ± 169 days (range 8–389 days) after the start of PP. Two patients (Cases 1 and 2) were treated with PP and rituximab concurrently within two weeks post-transplant. After a mean follow-up period of 22.5 months after rituximab, three children responded with complete remission (Cases 2, 3 and 4), but one (Case 4) relapsed within four months of remission. He received another dose of rituximab and currently remains on PP with improvement in proteinuria. One child (Case 1) had a partial response with a decrease in proteinuria, but it was not maintained. However, her kidney function has remained normal despite persistent nephrotic range proteinuria. No adverse side effects of treatment were reported. Patient characteristics, management, and outcome of the four cases are shown in Table 1.

**Case 1**

This female patient was diagnosed with biopsy-proven FSGS at the age of five years old. She progressed to end stage kidney disease within seven months and was started on hemodialysis. She received a deceased donor kidney transplant at the age of eight with immediate recurrence of the disease in the allograft. Despite seven months of PP, she eventually lost the allograft and was placed on peritoneal dialysis. Due to high sensitization to HLA-specific antibodies, she underwent a desensitization protocol with intravenous immunoglobulin. At age 13, she received a deceased donor kidney transplant and again had immediate recurrence of proteinuria. Laboratory findings on postoperative day 2 showed U_P/C_ 5.8, serum albumin 2 mg/dL, and serum creatinine 0.7 mg/dL. She was started on PP on postoperative day #2 and received the first dose of rituximab on postoperative day #10. A renal biopsy performed three months post-transplant showed extensive podocyte foot effacement suggestive of recurrence of FSGS. FSGS was confirmed on biopsy performed six months post-transplant with 20/25 glomeruli showing segmental sclerosis, varying from mild to global, with moderate to severe patchy interstitial fibrosis. She attained a partial response with a U_P/C_ nadir of 0.8 that was not sustained. PP was discontinued after 18 months. She currently has a U_P/C_ ratio of 1.8, but is clinically well with serum creatinine 0.65 mg/dL and serum albumin 3.1 mg/dL.

**Case 2**

This male patient was diagnosed with steroid resistant nephrotic syndrome at the age of 10 years old, which was later confirmed to be FSGS on biopsy. He was treated with cyclosporine and ACE inhibitor for six months with no response and progressed to end stage kidney disease and hemodialysis within three years. At age 13, he received a deceased donor kidney transplant. He had immediate recurrence of proteinuria with laboratory data of U_P/C_ 25–44, serum albumin 2.1 mg/dL, and serum creatinine 1.1. PP was started on postoperative day 5, and he received rituximab on postoperative day 14. He demonstrated good response to treatment within one month. Laboratory data revealed U_P/C_ 0.19, serum creatinine 0.9 mg/dL, and serum albumin 4.3 mg/dL. PP was discontinued 3 months post-transplant due to a central line infection. His current status 22 months post-transplant is U_P/C_ 0.1, serum creatinine 0.75 mg/dL, and serum albumin 4.2 mg/dL. He is maintained on lisinopril 10 mg daily.

**Case 3**

This female patient presented with nephrotic syndrome at 18 months of age. She was initially steroid sensitive and then became steroid resistant. Renal biopsy confirmed FSGS. She progressed to ESRD and was started on hemodialysis at age 14. She received a deceased donor kidney transplant at age 16. Nephrotic syndrome developed immediately post-transplant, and a transplant biopsy done on post-transplant day six showed extensive effacement of foot processes without focal sclerosis of the glomeruli (14 glomeruli). She was started on PP. The patient was PP dependent and received rituximab one year later. She went into complete remission and was weaned off PP. Currently 24 months post-rituximab, she has a Pr/Cr of 0.2 and serum creatinine 1.0–1.3 mg/dL.

**Case 4**

This young man presented with steroid resistant nephrotic syndrome at the age of five years old. FSGS was diagnosed on biopsy. Over a 12-year period, he was treated with multiple medications in an effort to induce remission. At age 17, his kidney function declined and he was placed on hemodialysis. After two months of dialysis, he received a deceased donor kidney transplant. The U_P/C_ ratio ranged 7–15.3 in the first post-transplant month, thought to stem from his native kidneys. Over the next two months, his U_P/C_ decreased to a nadir of 0.8 and serum albumin increased from 2.5 to 4 mg/dL. His U_P/C_ gradually began to increase over the following months up to 9.7, and serum albumin declined to 2.3 mg/dL. A biopsy of the allograft was performed seven months post-transplant. Results showed moderate to extensive effacement of the podocyte foot processes with absence of focal sclerosis of the glomeruli. PP was initiated three times a week with poor response, maximum U_P/C_ 26. The first dose of rituximab was administered 50 days after the start of PP. U_P/C_ decreased from 17.5 at the start of rituximab to 3.7 after the fourth dose. Nine months after the start of rituximab, the patient attained complete remission of proteinuria, U_P/C_ 0.18, on once a month PP, which was sustained for four months. Thirteen months after the start of rituximab, he relapsed with nephrotic range proteinuria, and PP therapy was intensified. There was no improvement in proteinuria over the following six months, max Pr/Cr 28. He received another dose of rituximab, 1 gram. B cell depletion was confirmed after the single dose and no further doses were given. Two months post-rituximab, the proteinuria improved, Pr/Cr 2.6 and PP were continued twice weekly.

## 4. Discussion

In this retrospective chart review, we found that three out of four cases (75%) with recurrent FSGS post-transplantation had a favorable response to the combination of rituximab and plasmapheresis with complete remission. One child who attained complete remission relapsed and subsequently received another dose of rituximab with improvement in proteinuria. One child demonstrated a partial remission of proteinuria, but it was not sustained. eGFR was stable in all patients, even in the child with persistent nephrotic-range proteinuria (Case 1). There were no adverse events related to rituximab such as infection or malignancy over the entire follow-up period in all four cases. 

Rituximab is a chimeric monoclonal antibody against the CD-20 antigen which results in depletion of B-lymphocytes. Its beneficial role in the treatment of nephrotic syndrome was discovered incidentally during treatment of idiopathic thrombocytopenic pupura in a patient with nephrotic syndrome [12] and in treatment of post-transplant lymphproliferative disorder in a pediatric renal transplant recipient with FSGS recurrence [13]. Since then, rituximab treatment for recurrent FSGS has been reported with mixed results. Yabu et al. described failure of rituximab used as a single agent in four adult transplant recipients [14]. However, in seven adults, the combined treatment of PP and rituximab induced complete or partial remission in 71.4% [15]. 

Pediatric patients may respond more favorably to rituximab [4]. A review of reported cases demonstrated a positive response to rituximab for the treatment of recurrent FSGS in 80% of pediatric transplant patients (Table 2) [4, 13, 16–21]. Of 15 cases (age 10.4 ± 4.5), complete remission was achieved in 10 (67%) and partial remission in 2 (13%). Three cases showed no response to treatment. In three cases, PP was discontinued prior to rituximab, and PP was not used at all in one case. Of note, although the average age (15.3 ± 2.6) in our series is higher than these reported cases, the outcomes are similar. A multicenter case series by Prytula et al. was not included in the review because information was obtained via survey, and repeat reporting of cases could not be ascertained. In this series of 15 pediatric transplant patients, 40% attained complete remission, 27% attained partial remission and 33% had no response to rituximab [22]. Data on PP was not available. 

Our series is unique in that it is the largest pediatric cohort of recurrence of FSGS with deceased donor allografts treated at a single center that has been described in the literature. Hickson et al. described a favorable outcome with rituximab in four pediatric patients who received living related donor allografts [4]. 

Our study is limited by the lack of consistent B cell monitoring in all cases. The child in Case 4 attained remission 9 months after an initial course of rituximab and relapsed after 13 months. The timing of B cell depletion and repletion in relation to these events is not known. B cell depletion was documented after a second round of rituximab, which was associated with reduction in proteinuria. However, as in other reported cases [4], the response to rituximab may be delayed by several months, and extended followup is required to determine responsiveness. 

Because of the variable time of initiation of rituximab in relation to PP, we cannot definitively attribute the successful outcome to a particular treatment. Rituximab is often used as rescue therapy after a trial of PP had failed to induce remission of proteinuria. Rituximab was given concurrently at the start of PP in two of our cases. Earlier administration of rituximab in conjunction with plasmapheresis may increase efficacy; however, the potential benefits require further investigation. It is important to note that even in the patients who had persistent proteinuria, the rituximab may have had a beneficial effect to stabilize GFR because one might have anticipated progressive decline in kidney function in these cases.

## 5. Conclusion

Rituximab is a safe and well-tolerated ancillary treatment for recurrent FSGS in pediatric patients in conjunction with PP. Multicenter clinical trials are needed to determine the efficacy of rituximab in this setting and to define the optimal timing, dose, and duration of this treatment.

## Figures and Tables

**Table 1:** Clinical features of cases of pediatric recurrent FSGS.

	Case 1	Case 2	Case 3	Case 4
Age at transplant	13	13	17	18
Sex	F	M	F	M
Ethnicity	Black	Asian	Hispanic	Hispanic
Donor	DD	DD	DD	DD
PP start (days posttxp)	2	5	6	217
PP duration (days)	540	93	485	527
Rituximab start (days posttxp)	10	14	395	267
Urine U_P/C_ Pre-PP	5.8	25–44	11	20
Urine U_P/C_ nadir	0.8	0.1	0.2	0.18
Urine U_P/C_ current	1.8	0.1	0.2	10.7
Current eGFR (mL/min/1.73 m^2^)	113	127	88	106
Follow-up postrituximab (months)	23	22	24	22
Current PP	No	No	No	Yes

F: female; M: male; DD: deceased donor; PP: plasmapheresis; U_P/C_: urine protein to creatinine ratio.

**Table 2:** Summary of the literature on use of rituximab in pediatric recurrent FSGS.

	Age	Sex	Donor	PP start (days posttxp)	Rituximab start (days posttxp)	Response	Notes
Nozu et al. [13]	12	M	LRD	N/A	186	CR	No PP given

Pescovitz et al. [16]	7	M	DD	14	155	PR	PP stopped 3 months prior to rituximab

Marks and McGraw [17]	6	M	DD	20	60	NR	PP stopped 3 months prior to rituximab
	10	M	DD	4	294	NR	Rituximab 750 mg/m^2^ × 2

Apeland and Hartmann [18]	18	M	DD	7	403	CR	

Bayrakci et al. [19]	14	M	LRD	−5	4	CR	PP started pre-txp

Dello Strologo et al. [20]	9	—	—	3629	3659	CR
	13	—	—	90	328	PR
	7	—	—	2	12	CR
	12	—	—	43	65	NR

Hickson et al. [4]	19	F	LRD	2	12	CR	
	6	M	LRD	−8	7	CR	PP started pre-txp
	13	M	LRD	−5	928	CR	PP started pre-txp
	5	M	LRD	−7	63	CR	PP started pre-txp

Grenda et al. [21]	5	M	DD	2	90	CR	PP stopped prior to rituximab

M: male; F: female; LRD: living related donor; DD: deceased donor; CR: complete response; PR: partial response; NR: no response; PP plasmapheresis.

*Does not include cases from Prytula et al. [22].

## References

[1] Andresdottir MB, Ajubi N, Croockewit S, Assmann KJM, Hibrands LB, Wetzels JFM (1999). Recurrent focal glomerulosclerosis: natural course and treatment with plasma exchange. *Nephrology Dialysis Transplantation*.

[2] Artero M, Biava C, Amend W, Tomlanovich S, Vincenti F (1992). Recurrent focal glomerulosclerosis: natural history and response to therapy. *American Journal of Medicine*.

[3] Hariharan S, Adams MB, Brennan DC (1999). Recurrent and de novo glomerular disease after renal transplantation: a report from Renal Allograft Disease Registry (RADR). *Transplantation*.

[4] Hickson LJ, Gera M, Amer H (2009). Kidney transplantation for primary focal segmental glomerulosclerosis: outcomes and response to therapy for recurrence. *Transplantation*.

[5] Pardon A, Audard V, Caillard S (2006). Risk factors and outcome of focal and segmental glomerulosclerosis recurrence in adult renal transplant recipients. *Nephrology Dialysis Transplantation*.

[6] Vincenti F, Ghiggeri GM (2005). New insights into the pathogenesis and the therapy of recurrent focal glomerulosclerosis. *American Journal of Transplantation*.

[7] Vinai M, Waber P, Seikaly MG (2010). Recurrence of focal segmental glomerulosclerosis in renal allograft: an in-depth review. *Pediatric Transplantation*.

[8] Baum MA (2004). Outcomes after renal transplantation for FSGS in children. *Pediatric Transplantation*.

[9] Cheong HI, Han HW, Park HW (2000). Early recurrent nephrotic syndrome after renal transplantation in children with focal segmental glomerulosclerosis. *Nephrology Dialysis Transplantation*.

[10] Garcia CD, Bittencourt VB, Tumelero A, Antonello JS, Malheiros D, Garcia VD (2006). Plasmapheresis for Recurrent Posttransplant Focal Segmental Glomerulosclerosis. *Transplantation Proceedings*.

[11] Pradhan M, Petro J, Palmer J, Meyers K, Baluarte HJ (2003). Early use of plasmapheresis for recurrent post-transplant FSGS. *Pediatric Nephrology*.

[12] Benz K, Dötsch J, Rascher W, Stachel D (2004). Change of the course of steroid-dependent nephrotic syndrome after rituximab therapy. *Pediatric Nephrology*.

[13] Nozu K, Iijima K, Fujisawa M, Nakagawa A, Yoshikawa N, Matsuo M (2005). Rituximab treatment for posttransplant lymphoproliferative disorder (PTLD) induces complete remission of recurrent nephrotic syndrome. *Pediatric Nephrology*.

[14] Yabu JM, Ho B, Scandling JD, Vincenti F (2008). Rituximab failed to improve nephrotic syndrome in renal transplant patients with recurrent focal segmental glomerulosclerosis. *American Journal of Transplantation*.

[15] Tsagalis G, Psimenou E, Nakopoulou L, Laggouranis A (2011). Combination treatment with plasmapheresis and rituximab for recurrent focal segmental glomerulosclerosis after renal transplantation. *Artificial Organs*.

[16] Pescovitz MD, Book BK, Sidner RA (2006). Resolution of recurrent focal segmental glomerulosclerosis proteinuria after rituximab treatment. *New England Journal of Medicine*.

[17] Marks SD, McGraw M (2007). Does rituximab treat recurrent focal segmental glomerulosclerosis post-renal transplantation?. *Pediatric Nephrology*.

[18] Apeland T, Hartmann A (2008). Rituximab therapy in early recurrent focal segmental sclerosis after renal transplantation. *Nephrology Dialysis Transplantation*.

[19] Bayrakci US, Baskin E, Sakalli H, Karakayali H, Haberal M (2009). Rituximab for post-transplant recurrences of FSGS. *Pediatric Transplantation*.

[20] Strologo LD, Guzzo I, Laurenzi C (2009). Use of rituximab in focal glomerulosclerosis relapses after renal transplantation. *Transplantation*.

[21] Grenda R, Jarmuzek W, Piatosa B Long-term effect of rituximab in maintaining remission of recurrent and plasmapheresis-dependent nephrotic syndrome post-renal transplantation—case report.

[22] Prytula A, Iijima K, Kamei K (2010). Rituximab in refractory nephrotic syndrome. *Pediatric Nephrology*.

